# Nested case control study of prevalence and aetiology of dementia in a rural Ugandan population, and a situational analysis of services available for affected families: a protocol. *Part of the DEPEND Uganda study (Dementia EPidemiology, unmet Need and co-Developing Solutions)*

**DOI:** 10.12688/wellcomeopenres.22944.1

**Published:** 2024-09-24

**Authors:** Josephine Prynn, Racheal Alinaitwe, Beatrice Kimono, Tunde Peto, Nicholas J Ashton, Claire J Steves, Joseph Mugisha, Martin Prince

**Affiliations:** 1 School of Life Course and Population Sciences, King's College London Faculty of Life Sciences & Medicine, London, England, UK; 2 MRC/UVRI and LSHTM Uganda Research Unit, Entebbe, Central Region, Uganda; 3 Makerere University School of Health Sciences, Kampala, Central Region, Uganda; 4 School of Medicine, Dentistry, and Biomedical Sciences, Queen's University Belfast, Belfast, Northern Ireland, UK; 5 Department of Psychiatry and Neurochemistry, University of Gothenburg, Gothenburg, Sweden; 6 Banner Health, Phoenix, Arizona, USA; 7 King's College London Institute of Psychiatry Psychology & Neuroscience, London, England, UK

**Keywords:** Dementia, Alzheimer’s, epidemiology, Africa, Uganda, prevalence, aetiology, biomarkers

## Abstract

**Background:**

The prevalence of dementia in low- and middle-income countries is increasing, yet epidemiological data from African populations remain scarce. Crucial risk factors differ in Africa from more intensively studied global areas, including a high burden of cerebrovascular disease and HIV, but lower rates of other risk factors like physical inactivity.

Understanding dementia aetiology in African settings has been limited by the expensive and invasive nature of biomarker testing. This study leverages developments in blood-based and retinal imaging biomarker technology to examine the drivers of dementia in older Ugandans.

People with dementia have complex needs benefiting from multi-dimensional support. Understanding current services will allow identification of barriers and opportunities to strengthen support available to people with dementia and their families.

**Methods:**

The study is nested within the existing General Population Cohort run by the MRC/UVRI & LSHTM Research Unit. Currently, all adults aged 60+ (around 1400) are undergoing brief cognitive screening.

In Part 1, cohort participants will be selected based on cognitive screening scores to undergo detailed cognitive assessment, using methods developed by the 10/66 Dementia Research Group.

Part 2 is a case control study of people with and without dementia using antecedent data, questionnaires, physical assessment, retinal imaging, and Alzheimer’s blood-based biomarkers. We will also compare disability, frailty, quality of life, and social engagement in people with and without dementia.

Part 3 assesses current provision of formal support for people with dementia through review of publicly available literature and expert interviews.

**Conclusions:**

This is the first study in Africa using blood-based and retinal imaging biomarkers to examine the pathological processes underlying dementia, and it will systematically map services available for people with dementia. This paves the way for effective policy strategies for both dementia prevention and support for people with dementia and their families.

## Plain language summary

**Background:**

The number of people experiencing dementia in low- and middle-income countries is increasing. However, there is little information from African countries examining how common dementia is and its causes. Data from high-income countries suggest that the most frequent brain changes linked to dementia are those associated with Alzheimer’s disease and blood vessel damage (cerebrovascular disease). Their identification has historically needed advanced scans and lumbar punctures which are very expensive and not always acceptable to people. This study uses new ways to examine brain changes including blood tests and photographs of the back of the eye (retina).

People with dementia have complex needs best addressed by a team of health and social care professionals. To strengthen this support, we need to understand the services currently available to people with dementia.

**Methods:**

This study is based within a repeated survey of a population in Uganda called the General Population Cohort (GPC), including around 1400 adults aged 60+. All participants will be given a brief memory test, and based on their scores, a subgroup will undergo a detailed assessment and an interview with someone who knows them well, to look for dementia.

We will compare people with and without dementia to look at dementia risk factors and brain changes using existing GPC data, questionnaires, physical assessments, blood tests and retinal photography.

Furthermore, we will be assessing the support currently available for people with dementia through a review of publicly available data and expert interviews.

**Conclusions:**

This will be first study in Africa to look for brain changes associated with dementia using blood tests and retinal photography, which will inform effective policy strategies for dementia prevention. We will also carry out the first systematic mapping of services available for people with dementia in Uganda: a crucial step towards strengthening them.

## Acronyms and abbreviations

AD                           Alzheimer’s Disease

ADNC                    Alzheimer’s disease neuropathological change

AVR                       Arteriole-to-venule ratio

CRAE                    Central retinal arteriolar equivalent

CRVE                    Central retinal venular equivalent

CSF                       Cerebrospinal fluid

CSID                     Community screening instrument for dementia

DBS                      Dried blood spots

GFAP                   Glial fibrillary acidic protein

GMS                    Geriatric mental state

GPC                     General Population Cohort

HAND                 HIV-associated neurocognitive disorder

HIV                     Human immunodeficiency virus

LMIC                  Low- and middle-income countries

MRI                    Magnetic resonance imaging

NFL                    Neurofilament light

NGO                   Non-governmental organisation

OR                      Odds ratio

PAF                     Population attributable fraction

PET                     Positron emission topography

p-tau                    Phosphorylated Tau

SD                       Standard deviation

SPPB                   Short physical performance battery

STRIDE               Strengthening Responses to Dementia

VaD                      Vascular dementia

WHO                   World Health Organisation

WOPS                  Wellbeing of Older People Study

## Introduction

Dementia has a profound effect on individuals, families, health systems, and societies and is a leading contributor to disability among older people in low- and middle-income countries (LMIC) ^1^. It is estimated by the World Health Organisation (WHO) that the number of people living with dementia will more than double globally between 2017 and 2050, mainly driven by increasing numbers in LMIC ^2^.

### Dementia prevalence data from African settings is scarce.

It is imperative to understand the scale of the burden of dementia to consider its impact on the population and how to address it. However, prevalence data from African settings remain scarce.

Early estimates of dementia prevalence in sub-Saharan Africa were from Ibadan, Nigeria in the 1990s, where prevalence was notably lower than in a comparable population of African-Americans in Indianapolis, USA ^3^. Since then, more data have emerged which were summarised in a systematic review and meta-analysis in 2017, showing that age-specific prevalence of dementia appeared comparable to prevalence figures across global regions, with 1.7% of people aged 60–64 experiencing dementia, rising to 23.3% of people aged 95+ ^4^. The review included all population-based studies in adults aged 60+ within sub-Saharan Africa and found 12 studies undertaken in 6 countries.

Since then, a population study of 400 adults over 60 in South-West Uganda in 2020 estimated that 20% had dementia ^5^. Participants were assessed using the Brief Community Screening Instrument for Dementia (Brief CSID), but no detailed cognitive testing or diagnostic assessment was done. The authors used previously published sensitivity and specificity data of the screening tool derived from populations in Latin America, China, India, and Nigeria ^6^ to back-estimate the likely dementia prevalence. In Northern Uganda in 2023, another study also used the Brief CSID in a population-based survey of 434 adults over 50, and estimated a prevalence of 23% of possible or probable dementia ^7^. These estimates, based solely on brief screening tools, are higher than expected based on other regional data ^4^ Further investigation using in-depth cognitive, psychological and physical assessments is crucial to explore these findings, and understand the underlying drivers of dementia in Ugandan populations.

### Risk factors and aetiology of dementia may differ in African populations compared to other global regions.

***Dementia Risk Factors***

Fourteen key potentially modifiable risk factors have been identified for all-cause dementia: low educational attainment, midlife hypertension, midlife obesity, hearing loss, smoking, depression, physical inactivity, social isolation, diabetes, air pollution, excessive alcohol consumption, traumatic brain injury, high low-density lipoprotein cholesterol and untreated vision loss ^8^. Together these factors have been estimated to cause around 45% of worldwide dementia cases. However, this is based on data mainly from high-income settings, and assumes both similar strengths of association between individual risk factors and dementia, and prevalence of each risk factor in less comprehensively researched populations. While studies in African settings have demonstrated links between lower educational attainment, vascular risk factors, and social isolation with dementia ^5, 9– 13^, data is minimal on other risk factors particularly midlife obesity, alcohol, depression, hearing loss and traumatic brain injury.

***Pathological processes leading to dementia***

Most people with dementia have multiple contributory pathological processes. Of these Alzheimer’s disease neuropathological change (ADNC) is the process with the highest prevalence, followed by vascular changes ^14^.

Alzheimer’s disease neuropathological change (ADNC) is characterised by the presence of amyloid and phosphorylated tau (p-tau) in the brain, which when associated with clinical dementia is Alzheimer’s disease (AD). AD is considered a progressive neurodegenerative condition which is 60–80% heritable, particularly through the APOE4 genetic pathway ^15^, and ADNC changes start many years before symptom onset ^15^. People with ADNC but without clinical dementia are sometimes considered in a pre-dementia stage ^16^, but the progression to dementia within a given timeframe is not guaranteed, and the factors influencing resilience to these changes are not yet well understood ^14^. Validated biomarkers for ADNC used in clinical and research settings in high-income countries include characteristic magnetic resonance imaging (MRI) findings, amyloid-PET and tau-PET scanning, and CSF-biomarkers for amyloid β1–40, amyloid β1–42, phosphorylated-tau 181 and total tau ^15^.

Dementia associated with vascular changes, including infarcts, small vessel disease, and micro-bleeds secondary to amyloid angiopathy is known as Vascular Dementia (VaD) ^17^. Risk factors align with other vascular pathological processes, including smoking, hypertension, diabetes, and hypercholesterolaemia. Diagnosis is supported by signs of cerebrovascular disease on MRI ^17^. There is increasing recognition that ADNC and cerebrovascular disease commonly co-exist, particularly in the oldest age groups ^14^, and may synergistically impact cognition ^18^.

In the pre-antiretroviral era, HIV-associated neuro-cognitive disorder (HAND) was commonly seen with HIV infection ^19^. However, understanding whether chronic and well-controlled HIV impacts cognition with ageing is challenging, due to difficulties disentangling the effects of comorbidity and ART ^20^, and a paucity of studies in older people. However, HIV infection has been linked to accelerated biological ageing ^21^, increased incidence of Alzheimer’s disease ^22^, and vascular complications ^23^.

Little is known of dementia drivers within Africa, as studies characterising dementia subtype have relied on clinical criteria ^24– 26^, which have poor diagnostic accuracy compared to neuropathological examination, the gold standard ^27, 28^. From limited data, it is suggested that cerebrovascular disease may be a greater contributor to dementia in Africa than other global regions ^29, 30^. This is plausible due to a high burden of stroke seen in many African settings ^31^, and a high prevalence of undiagnosed and untreated vascular risk factors ^32, 33^. This urgently needs clarifying as it implies that more dementia is preventable with adequate control of vascular risk factors. Furthermore, the extent of genetic vulnerability to developing AD as mediated by the APOE4 allele is less clear among individuals of African ancestry than those of European ancestry. This is poorly understood due to the scarcity of available genomic data from African populations and wide genetic diversity within the continent ^29^.

This study takes advantage of recent scientific advances in biomarkers for ADNC and cerebrovascular disease allowing identification of these processes, without the need for prohibitively expensive or invasive neuroimaging and CSF biomarker analysis ^16^.

### Novel diagnostic biomarkers enable identification of pathological processes leading to dementia

***Blood based biomarkers for Alzheimer’s disease neuropathological change***

Thanks to the development of new high-sensitivity assays, biomarkers for ADNC previously only accessible in cerebrospinal fluid (CSF) are now detectable in blood at much lower concentrations ^34^. Amyloid beta and phosphorylated tau (p-tau) concentrations are highly correlated with the well-established CSF and PET biomarkers of ADNC, while neurofilament light (NfL) and glial fibrillary acidic protein (GFAP) are informative biomarkers of neurodegeneration ^34^. Using a panel of biomarkers will enable us to identify participants with ADNC, and distinguish AD from other neurodegenerative processes in participants with clinical dementia. Furthermore, recent technological advances have enabled identification of biomarkers from dried blood spots (DBS). This is a highly promising option where access to cold-chain for sample storage and transportation is limited or lacking, ^35^.

***Retinal imaging for cerebrovascular disease***

Changes in the retinal vasculature are very highly correlated with cerebral vasculature, due to shared anatomical and physiological properties of the brain and retina ^36^. Recent advances in semi-automated and automated analysis of retinal vascular morphology allow retinal imaging to act as a proxy for direct brain imaging to identify cerebrovascular disease ^37– 39^. The measures of interest are retinal vessel calibre changes, specifically the central retinal arteriolar equivalent (CRAE), central retinal venular equivalent (CRVE), and their relationship – the arteriolar-venular ratio (AVR) - as well as quantification of tortuosity and fractal dimension of vessels ^40^. This is a highly novel and promising option for identification of cerebrovascular disease in remote or low-income settings.

### People living with dementia in Uganda have unmet health and social care needs.

Dementia is among the most important causes of disability and care needs for older people in LMIC ^1, 41– 43^, and is associated with stigma and social isolation ^29, 44^. People experiencing dementia have complex needs that benefit from multi-dimensional support including medical input for diagnosis, support with advanced care planning, caregiver support, and therapeutic interventions to promote ongoing independence ^45, 46^.

However, there is a significant gap between this demand and the provision in many LMIC, including in Africa ^47^. Qualitative work with healthcare providers in southwestern Uganda showed that while clinicians with specialist mental health training felt confident in diagnosing and managing dementia, many other healthcare workers felt ill-equipped to assess people with dementia, while some believed that dementia symptoms were a normal part of ageing ^48^.

Caregivers of people with confirmed dementia in Uganda reported multiple contacts with healthcare prior to receiving a diagnosis, and most had presented to healthcare for related problems (such as pressure ulcers or seizures) rather than the cognitive impairment. They also described the high cost of medication and investigations, requiring selling of assets ^49^. Caregivers reported feeling unsupported in understanding how best to care for the person with dementia ^50^. Understanding the conformation and function of current services will allow identification of barriers and opportunities for strengthening the support available to people with dementia and their families.

This study will be partnered with a study investigating the experiences and unmet needs of people with dementia and their caregivers within the same community using qualitative methods.

## Aims and objectives

### Aim

To increase understanding of the epidemiology of dementia in Uganda, and work towards a strategy for risk reduction and improving outcomes and care.

### Objectives

Within a rural Ugandan population to:

1) Evaluate the prevalence of dementia.
  a. What is the prevalence of dementia in adults aged 60 and over within one rural Ugandan population?
  b. What is the prevalence of dementia among people aged 60 and over living with HIV?
  c. What is the sensitivity and specificity of the WHO-recommended cognitive screening tools for dementia?
2) Establish the causes and associated factors of dementia to inform risk reduction strategies.
  a. What is the association of socio-economic position, education, cardiovascular risk factors, and HIV with dementia?
  b. What is the relative importance of Alzheimer’s disease compared to cerebrovascular disease and other dementia causes in this setting?
  c. Can venous and capillary DBS be used to accurately assess Alzheimer’s disease biomarkers in a rural Ugandan setting?
3) Understand the formal support available for people living with dementia and their families.
  a. What medical diagnostic and treatment services are available and accessible for people living with dementia?
  b. What social care and support services are available for people living with dementia and their families?
  c. What financial support is available for people living with dementia and their families?

## Protocol

### Study design

There are three parts to this study.

**Part 1**: Cross-sectional survey to understand the prevalence of dementia.

**Part 2**: Nested case control study to understand the risk factors and underlying pathological processes leading to dementia and its associated factors, with a cross-sectional analysis frailty, disability, and quality of life of older people with and without dementia.

**Part 3**: Situational analysis of the formal care and support context in which people with dementia and their caregivers are living. This will include:

1) A desk review of information available in the public domain.
2) Qualitative semi-structured interviews with dementia experts.

### Study setting

The study will be nested within the existing **General Population Cohort (GPC)** at MRC/UVRI & LSHTM Uganda Research Unit (MUL). This is an open cohort that was established in 1989 with follow-up every two years, and comprises all residents of a sub-county of Kulungu District ^51^. At the last census round in 2021–2023, there were around 1400 adults aged 60+. Data are routinely gathered on socio-demographics, medical history, lifestyle, anthropometry, blood pressure, and HIV status. At the current round of data collection, which started March 2024, for people aged 60 and over the data collected includes cognitive screening, which is performed using WHO-recommended tools:

- Verbal fluency: number of animals named within one minute.
- Immediate and delayed verbal recall of 10 words.
- Digit span: recalling increasing numbers of digits forwards and backwards.

We will use existing cognition data from the **Wellbeing of Older People Study (WOPS)** to estimate local population norms for these screening tests. WOPS is a population-based cohort enriched for HIV+ participants that was established in 2009 and includes participants from Kulungu District as well as from Masaka town and Wakiso District (near Entebbe). It comprises around 500 adults over 60, of whom 50% were HIV+ at the time of recruitment ^52^. In addition to socio-demographic and health questionnaires, cognitive screening is performed using the WHO-recommended tools that have recently been introduced into the GPC. As the WOPS population is not population-representative, the mean and standard deviations (SDs) will be adjusted to the socio-demographic distribution of the underlying population using inverse probability weighting. 

### Part 1 – Cross-sectional survey to understand the prevalence of dementia

***Population***

The study population will be adults aged 60+ from the GPC cohort, selected based upon their cognitive screening results. We aim to recruit all of those with impaired cognition at screening (defined as ≤1.5 SDs below the mean), a random sample of 50% of those with mildly impaired cognition (≤1 to <1.5 SD below the mean), and a random sample of 15% of those with unimpaired cognition (>1 SD below the mean). We will ask the older person to identify an informant: someone who knows them well and if relevant, cares for them, to share their experience of the older person’s cognition and behaviour.

***Sample size***

Around 300 older people will have in-depth cognitive assessment based on their performance at screening tests. In view of the two-phase sampling design, the precision of the prevalence estimate will depend on the sensitivity and specificity of the screening tools, which is yet to be determined.

***Procedures***

Participants will be recruited on a rolling basis while GPC data collection is ongoing, based on their score at cognitive screening. 

Older people and informants will be approached to participate in their homes by Luganda-speaking interviewers. The purpose of the study will be explained and informed consent sought. More detail on consent procedures can be found in the Ethical Considerations section. Participants will also be asked at this stage whether they would want to be informed of a dementia diagnosis if it was made based on these assessments.

If participants are not at home at the time of the visit, we will make at least 2 further attempts to contact them at different times of day.

 Their cognitive assessment will be done using the following three tools:

**1)**  **Community Screening Instrument for Dementia (CSI-D)**

The includes assessment of cognition across multiple cognitive domains, and an informant account of the older person’s cognition and function, including whether there has been any deterioration in cognition. It was developed specifically for use in diverse lower-literacy populations, and has been used across the world in multiple settings, including in Africa ^53^.

**2)**  **Modified Consortium to Establish a Registry of Alzheimer’s Disease (CERAD) 10-word list-learning test with delayed recall**

This is a widely used tool for assessing cognition and has shown minimal bias across different cultural contexts ^54, 55^.

**3)**  **Geriatric Mental State (GMS)**

This assesses the older person for clinically significant mood disorders (depression and anxiety), and psychosis as well as cognition, integrating this information algorithmically to generate a probable principal diagnosis.

Combining the output of these three measures to generate a diagnosis of probable dementia was pioneered by the 10/66 Dementia Research Group, who developed an algorithm validated against psychiatrist diagnosis of dementia in multiple global regions including Africa ^56^. The methods have been extensively used across the world, and have shown good predictive validity in cohort follow-up ^57^. As these tools have not previously been used in Uganda, we will review the tools with the study advisory group prior to starting data collection, and pilot rigorously with older people from the WOPS cohort to ensure the tools are acceptable and appropriate to this setting, as described later.

The whole assessment will take between 50 and 90 minutes, depending upon the extent of morbidity, and any communication difficulties. Interviews will be held in a quiet private space where possible, for both confidentiality of the participants, and, by avoiding distractions, to maximise their performance in cognitive testing.

***Analysis plan***

Dementia prevalence will be presented as the percentage of the GPC population with dementia, with 95% confidence intervals ^58^. This will be calculated using weighted logistic regression accounting for the two-phase sampling methodology ^59^.

The WHO-recommended screening tools will be validated against a diagnosis of dementia, calculating sensitivity, specificity, and positive and negative predictive values in this population.

### Part 2 – Case control study to understand the risk factors and pathological processes leading to dementia, with a cross-sectional comparison of disability, frailty, and quality of life among people with and without dementia. 

***Population***

**Figure 1.**
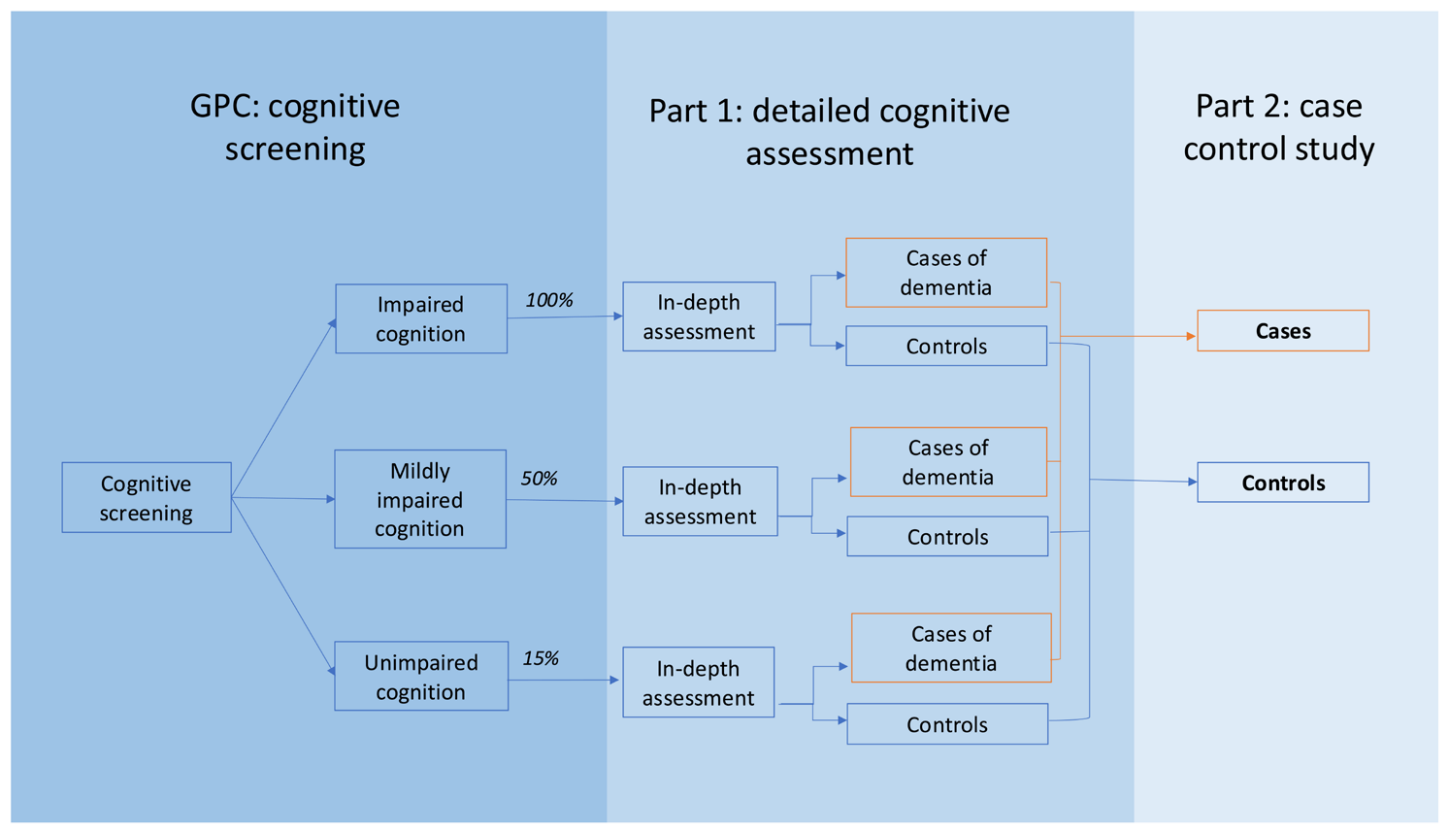
Outline of the stages of recruitment for Part 2 of the study.

We will aim to recruit all participants who have been identified to have dementia in Part 1 as cases. Controls will be all GPC participants who have undergone detailed cognitive assessment at Part 1 who do not have dementia. This is summarised in Figure 1.

***Sample size***

Recruitment of cases will continue until all scheduled Part 1 assessments have been completed. The total number will therefore depend on the underlying prevalence, the attrition rate of participants between the different study parts, and the sensitivity and specificity of the screening tools. There will be up to four controls per case, depending on the number of cases identified. If prevalence of dementia or participation rates are low yielding few potential cases, we will also recruit participants with low scores at cognitive screening from the WOPS cohort who would then go through the same cognitive assessment procedures, aiming for at least 80 cases in total. If unexpectedly high numbers of people with probable dementia are identified, recruitment for the case control study will stop once 200 cases have been identified. Controls would then be selected only from the villages within the GPC population that yielded the cases.

In Table 1, the detectable odds ratio (OR) with 80% power and 95% confidence are outlined for different exposure prevalence levels among controls and an assumed 80 cases of and two controls per case.

**Table 1.** Power calculation for case control study in Part 2.

Number of cases recruited	Control : Case ratio	Prevalence of exposure among controls	Detectable OR
**80**	2	50%	2.2
**80**	2	20%	2.3
**80**	2	6%	3.3

***Inclusion and exclusion criteria***

Cases will be identified based on their assessment in Part 1. To further ensure that these cognitive assessment tools are accurately identifying dementia, at least the first 25 cases identified will also undergo a clinical review by a doctor experienced in dementia diagnosis. This review will be done at participants’ homes. If concordance between clinical review and the 10/66 tools is high, then the remaining cases will be identified using the 10/66 tools alone. Otherwise, all potential cases will undergo a clinical review to confirm eligibility as cases prior to allocation to the case control study.

***Procedures***

Eligible participants will be approached at home to invite them for Part 2, which will be completed at the Kyamulibwa Research Station. Informed consent will be sought, using the same processes as in Part 1. The key exposures in the case control study are summarised below.

- Key risk factors
  - Educational attainment
  - Vascular risk factors
  - History of head injury
  - Alcohol excess
  - HIV
  - Social isolation
  - Hearing loss
  - Household air pollution
- Pathological processes
  - Alzheimer’s neuropathological change (using blood biomarkers)
  - Cerebrovascular disease (using retinal imaging)

The following data will be collected:

**Questionnaires**

Questionnaires for both the older person and their informant will be administered by interviewers in Luganda.

The questionnaire for participants will include the following elements:

- **Socio-demographic information** – study-specific questionnaire.
- **Dementia risk factors** – study-specific questionnaire covering alcohol intake, early life adversity, vision, hearing, and medical history.
- **Disability** – WHO Disability Assessment Schedule (WHODAS) 2.0 (12-item) ^60^.
- **Social engagement** – study-specific questionnaire adapted from the WOPS study ^61^.
- **Quality of life in dementia** – DEMQOL ^62^.

It is likely that some participants will be unable to reliably answer questionnaires due to cognitive impairment and will require an informant to answer a proxy version of the questionnaire. However, cases and controls must undergo identical procedures to minimise information bias, and interviewers will be masked to whether the participant is a case or a control. Therefore, this approach will not be restricted to cases only: for all participants, informants will also be administered proxy versions of all the older person’s questionnaires. At the end of each interview with both the older person and the informant, the interviewer will be asked to subjectively rate their confidence in the reliability of the answers given. Where there is doubt in the reliability of the older person’s answers, regardless of whether they are a case or control, and the informant’s answers were perceived as more reliable, the informant’s answers will be used in place of the older person’s answers for the analysis.

Older people should be assisted as much as possible in completing the assessments, including ensuring the use of any eyeglasses or hearing aids that they usually use, and taking breaks as necessary. Communicator headphones will be provided for people with uncorrected hearing impairment.

The questionnaire specifically for informants will include the following elements:

- **Socio-demographic information**: data from previous GPC waves, study-specific questionnaire
- **Care needs of the older person**: study-specific questionnaire adapted from the WOPS study ^61^
- **The older person’s social participation**: IDEA-IADL ^63^.
- **Depression and anxiety:** PHQ-9 ^64^ GAD-7. ^65^,
- **Quality of life**: WHOQOL-Bref ^66^.
- **Presence of behavioural and psychological symptoms of dementia in the older person**: Brief Neuropsychiatric Inventory Questionnaire (NPI-Q) ^67^.
- **Impact of supporting the older person** (informants identifying as caregivers only): Zarit Burden interview ^68^.

**Physical assessment**

Physical assessment of older people will be done using an adapted version of the established NEUROEX tool ^58^ and includes the following elements:

- Structured neurological examination.
- Gait speed – participants will be timed walking 4 metres.
- Brief dental examination.
- Hearing – using the WHO-recommended Whispered Voice Test.
- Distance vision – using the PEEK application on tablet computers or Tumbling E chart.
- Grip strength

Furthermore, to complete the required elements of the Short Physical Performance Battery (SPPB) ^69^, we will do the following assessments:

- Chair stand test – the time taken to complete five sit-to-stands.
- Balance test – participants will be timed to test if they can stand for 10 seconds with their feet together, feet in a semi-tandem position, and feet in a full-tandem position with their eyes open.

**Blood tests**

Blood tests will be done for both cases and controls to look for reversible causes of dementia:

*Set 1:*

- Full blood count – and if anaemia is present, Vitamin B12.
- Creatinine.
- Thyroid Stimulating Hormone.
- HbA1C.
- Syphilis screening with Rapid Plasma Reagin – if positive, treponemal testing.

*Set 2:*

Blood will be taken for blood-based Alzheimer’s biomarkers:

- Amyloid beta 42 and 40.
- Phosphorylated Tau-217.
- Neurofilament light.
- Glial fibrillary acidic protein.

This involves both a venous blood sample for all four blood-based biomarkers and capillary finger-prick samples for dried blood spots for p-Tau217, NfL, and GFAP.

**Retinal imaging**

Participants will have retinal photography done of each eye using a 3nethra Classic camera to assess the vasculature. Images will be interpreted using automated processes to quantify vascular retinal morphology, including the central retinal arteriolar equivalent (CRAE), central retinal venular equivalent (CRVE), arteriolar-venular ratio (AVR) ^39^.

***Analysis plan***

We will use unconditional multivariable logistic regression to calculate odds ratios for each exposure of interest. This will be adjusted for possible confounders, and account for any clustering at household level, and sampling probabilities in Part 1 of the study.

We will use these data to calculate the population attributable fraction (PAF) for each individual risk factor and overall. The PAF for the pathological processes (Alzheimer’s pathology and cerebrovascular disease) will indicate the relative importance of these two processes in the development of clinical dementia in the population studied.

For people with and without dementia, we will compare the older person’s quality of life, social engagement, disability, frailty, and quality of life.

We will assess the correlation and agreement between the AD biomarker values in EDTA plasma samples with dried blood spot samples using Spearman rank correlation and intra-class correlation coefficients. 

### Part 3 - Situational analysis of the formal care and support context in which people with dementia and their caregivers are living.

***Population***

In addition to a desk review we will be recruiting key decision-making and topic expert stakeholders for interviews. Potential participants will be approached from the following sectors: health and social care, civil society groups and non-governmental organisations, traditional healing, and government officials, at both regional and national levels, and across geographic regions. They will need to have at least 2 years of experience within their sector to participate. We will identify potential participants through publicly available information on websites and through snowballing methods.

***Procedures***

We will be using an adaptation of the STRIDE (STrengthening Responses to DEmentia) analysis tool ^70^ to undertake a thorough situational analysis of the formal care and support landscape in which people with dementia are living in Uganda. This has been developed to allow researchers to gather comparable data across different settings and has already been used in South Africa and Kenya ^47, 71^, among other countries.

1) Desk review

This is a review of data in the public domain to provide a summary of the health system, long-term care system and policy contexts relevant to dementia, as well as dementia awareness and stigma, and social protection for people with dementia specific to Uganda. This is summarised in Table 2.

**Table 2.** Summary of the domains covered in the desk review as part of the situational analysis.

Domain	Contents
**Overall country context**	Population and demographic characteristics Epidemiological situation Economic and social situation Social protection Political situation
**Overall health system context**	Health system organisation Health system financing Health system workforce
**Overview of the long-term care system context**	Long-term care system organisation Long-term care system financing Long-term care workforce
**Dementia policy context**	Governance Dementia policies and plans Legislation Clinical guidelines, standards and protocols for dementia Dementia care co-ordination The policy process
**Dementia awareness and stigma**	Public awareness and education
**Epidemiology and information systems for dementia**	Epidemiology Information systems
**The dementia care system**	Overview of the dementia care system Dementia care system organisation Dementia care system workforce Health and long-term care facilities Antidementia medication and care products
**Unpaid care and other informal care for dementia**	Informal care workers Family/unpaid care
**Social protection for people with dementia**	
**Dementia research**

2) Interviews with experts

This stage involves in-depth interviews on the emerging findings, to gain a more thorough understanding of the experiences of people with dementia and their communities, and the potential routes to developing or scaling-up an evidence-informed care pathway. We will start with around ten participants and increase numbers until saturation is reached.

Interviews will explore what health services are available for people with dementia, identification and management of dementia, awareness and prevention, long-term care services, policy prioritisation, and elder abuse. They will make use of vignettes, initially developed by the STRIDE team ^70^, to represent cases of people developing dementia symptoms in different contexts in Uganda. The aim is to prompt discussion on the likely or possible patient journeys and services available, to identify both the barriers to diagnosis and support, and any examples of best practice.

***Analysis plan***

Interviews will be digitally recorded and transcribed verbatim. Transcripts will be entered into NVivo software for analysis. We will use a framework analysis approach to code the interview data by pre-defined themes identified through the desk review.

### Stakeholder engagement

We will establish a study advisory group including members of civil society groups such as the Uganda Alzheimer’s Association, Uganda-based researchers and/or clinicians with dementia expertise, and older people with and without dementia. We will engage with this group throughout the project, seeking advice and support regarding community-sensitisation, data collection tool development, supporting people diagnosed with dementia through the study, and dissemination of research output.

### Translation and piloting

All data collection tools will be translated from English to Luganda using translation/back-translation methods. That is, a translator proficient in English and fluent in Luganda will translate the tools into Luganda. An independent translator proficient in Luganda and fluent in English will back-translate the tools to English. Discrepancies arising between the original tool and the translated/back-translated version will be discussed with both translators to find a consensus. The translated version will then be discussed with the study advisory group.

For the piloting, we will recruit participants and informants from the WOPS cohort with a broad range of scores at cognitive screening at most recent testing, and who are not also in the GPC, as there is some overlap. This will be to check a) how well the questions are understood by participants (including the use of cognitive interviewing techniques), and b) practical elements of the data collection including the duration of the assessments.

### Informed consent processes

Participants will be required to give informed consent prior to data collection. It is important for this study that people without the capacity to consent are not excluded, as people with dementia have the right to contribute to research that may help them or other people with dementia in the future. For participants lacking capacity to consent participation will be agreed with their closest relative or carer and documented on an Informant Declaration Form. If the participant gives any verbal or non-verbal indication of disagreeing with any part of the study, then they will not be included.

Consent will be documented by signing a consent form for those able to write, or a thumb print for those who are not. For people unable to read, this will be witnessed and signed by another community member who is able to read.

### Dissemination of findings

Integral to this study is active engagement with the local community and wider scientific and policy-making groups. Our dissemination plan will be made in close collaboration with the GPC Community Advisory Board and the DEPEND Study Advisory Group.

Throughout and following the study, we will hold regular meetings with the GPC Community Advisory Board to update them about the study and publicise its main outcomes. Additionally, we will communicate directly with community members through meetings organised with the support of village leaders.

We plan to communicate the findings of this study with policy makers, researchers, and implementers through the production and dissemination of open-access academic papers in high-impact publications, timely newsletters, and policy briefs. We will invite decision-making and policy stakeholders relevant to dementia and older people’s health to a workshop to disseminate the main findings with a focus on the implications for public health and policy.

### Data management plan

***Questionnaires***

The questionnaires will be programmed onto encrypted tablet computers using ODK. This is linked to a central database from previous rounds of data collection, to allow easy identification of participants. After collection, the data will be downloaded from the tablet computers to secure databases on a local project server daily. Data will be regularly backed up and stored offsite on secure servers, without identifiable details. Identifiable details will be held securely by the MUL data management team, and will only be accessed if participants need to be contacted for clinical reasons.

***Retinal images***

Retinal images will be stored confidentially in encrypted files, labelled with participant ID numbers only. Images and other relevant data (e.g. age, sex, visual acuity) will be shared with external collaborators for analysis will include no identifiable information.

***Laboratory Data***

Samples will have initial processing at the laboratory at the Kyamulibwa Research Station, then transferred in batches to the MRC-Uganda laboratory in Entebbe for testing.

Samples taken for AD biomarkers will be stored in aliquots, frozen, and subsequently shipped to Sweden for analysis. The freezing facility has a back-up generator to ensure no loss of power and is monitored using an alarm system. Samples sent to remote laboratories for analysis will not have any participant identifiable details, and the process will be covered by a Material Transfer Agreement.

***Qualitative data***

Interviews will be recorded on a digitally encrypted Dictaphone, and files will be uploaded as soon as possible onto KCL OneDrive labelled with participant ID numbers. The audio file will then be deleted from the Dictaphone. The files used for transcription and translation from Luganda to English will include no identifiable details, but it is possible that the audio content could contain information suggestive of the interviewee’s identity. Therefore, any third party involved with transcription or translation will be subject to a strict Data Sharing Agreement to maintain interviewees’ confidentiality. Transcript documents will be anonymised and labelled with only broad category of interviewee (e.g. age, sex, role) to give context to their data.

***Data sharing***

MRC-Uganda is committed to open access data and publications. The data generated will be suitable for sharing as it will be anonymised and may be relevant to a range of different research questions. The Data Sharing Policy will be in line with the Wellcome Trust Policy on Data Management and Sharing.

Once the data sets have been finalised, they will be archived and stored with no identifiable information on the King’s Research Data Repository, KORDS for a minimum of 10 years. They will be available to access after findings have been published in an academic journal, and will be accompanied by a codebook metafile providing an accurate description of the content. The concept of data sharing will be included on the participants’ consent forms to ensure we have their broad and enduring consent. The Data Sharing Policy will specify that the researchers do not attempt to identify study participants from released data or breach confidentiality in any way, or to make unapproved contact with study participants.

Study protocols, SOPs, and data collection tools will be made freely available after completion of the study.

## Impact of the study and future work

This study will provide evidence of the scale and impact of dementia in this population. Understanding the unmet needs of people with dementia and their carers is the first step in motivating and supporting local, national, and international organisations, and healthcare providers to reach this population.

Furthermore, modelling of future dementia prevalence and public health planning for dementia prevention requires robust data on the causes and drivers of dementia in a specific population, which this study will provide.

This will be the first study to use retinal vessel calibre interpretation and among the first using Alzheimer’s disease blood biomarkers in Africa. By extending these non-invasive and hugely valuable techniques into the region, opportunities will be created for much more detailed and accurate research into not only dementia causes and drivers across the continent, but also cardiovascular disease more generally.

Sensitisation about dementia within the community throughout this project will increase community understanding of dementia as a medical condition, which may cause attitudinal change and increased support for people with dementia and their carers.

This study will pave the way for further work in multiple domains. For example, follow-up of participants would enable assessment of the predictive validity of the dementia diagnostic tools used, as well as prognosis of participants with dementia, incidence of dementia, and future prevalence of dementia. It also provides an ideal platform for interventions, both to support people with dementia and their caregivers, and for participants identified as at high risk of developing dementia in the future. 

## Ethics and consent

We have been granted ethical approval from the Uganda Virus Research Institute Research Ethics Committee (GC/127/1023) dated 19 ^th^ July 2024 and King’s College London Research Ethics Committee (HR/DP-23/24-42211) dated 30 ^th^ July 2024. Prior to starting data collection we will also ensure that we have approvals from the Uganda National Council of Science and Technology and the London School of Hygiene and Tropical Medicine Research Ethics Committee. 

Participants will be required to give informed consent prior to data collection. It is important for this study that people without the capacity to consent are not excluded, as people with dementia have the right to contribute to research that may help them or other people with dementia in the future. For participants lacking capacity to consent participation will be agreed with their closest relative or carer and documented on an Informant Declaration Form. If the participant gives any verbal or non-verbal indication of disagreeing with any part of the study, then they will not be included.

Consent will be documented by signing a consent form for those able to write, or a thumb print for those who are not. For people unable to read, this will be witnessed and signed by another community member who is able to read.

## Data Availability

No data are associated with this article.

## References

[1] Prince MJ Wu F Guo Y : The burden of disease in older people and implications for health policy and practice. *Lancet.* 2015;385(9967):549–562. 10.1016/S0140-6736(14)61347-7 25468153

[2] World Health Organisation: Global status report on the public health response to dementia.Geneva,2021. Reference Source

[3] Hendrie HC Osuntokun BO Hall KS : Prevalence of Alzheimer's disease and dementia in two communities: Nigerian Africans and African Americans. *Am J Psychiatry.* 1995;152(10):1485–92. 10.1176/ajp.152.10.1485 7573588

[4] Wimo A Aboderin I Guerchet M : Dementia in sub-Saharan Africa: challenges and opportunities.ed. A.s.D. International. London,2017. Reference Source

[5] Mubangizi V Maling S Obua C : Prevalence and correlates of Alzheimer’s disease and related dementias in rural Uganda: cross-sectional, population-based study. *BMC Geriatr.* 2020;20(1): 48. 10.1186/s12877-020-1461-z 32041525 PMC7011370

[6] Prince M Acosta D Ferri CP : A brief dementia screener suitable for use by non-specialists in resource poor settings—the cross-cultural derivation and validation of the brief community screening instrument for dementia. *Int J Geriatr Psychiatry.* 2011;26(9):899–907. 10.1002/gps.2622 21845592 PMC3427892

[7] Benyumiza D Kumakech E Gutu J : Prevalence of dementia and its association with central nervous system infections among older persons in Northern Uganda: cross-sectional community-based study. *BMC Geriatr.* 2023;23(1): 551. 10.1186/s12877-023-04174-9 37697266 PMC10496337

[8] Livingston G Huntley J Liu KY : Dementia prevention, intervention, and care: 2024 report of the Lancet standing commission. *Lancet.* 2024;404(10452):572–628. 10.1016/S0140-6736(24)01296-0 39096926

[9] Ikanga J Reyes A Kaba D : Prevalence of suspected dementia in a sample of adults living in Kinshasa-Democratic Republic of the Congo. *Alzheimer's Dement.* 2023;19(9):3783–3793. 10.1002/alz.13003 36880714 PMC10483015

[10] Akinyemi RO Allan L Owolabi MO : Profile and determinants of vascular cognitive impairment in African stroke survivors: the CogFAST Nigeria study. *J Neurol Sci.* 2014;346(1–2):241–9. 10.1016/j.jns.2014.08.042 25238666

[11] Gureje O Ogunniyi A Kola L : Incidence of and risk factors for dementia in the Ibadan study of aging. *J Am Geriatr Soc.* 2011;59(5):869–874. 10.1111/j.1532-5415.2011.03374.x 21568957 PMC3173843

[12] Hall K Murrell J Ogunniyi, A : Cholesterol, APOE genotype, and Alzheimer disease. *Neurology.* 2006;66(2):223–227. 10.1212/01.wnl.0000194507.39504.17 16434658 PMC2860622

[13] Mworozi K Ameda F Byanyima RK : Carotid artery plaque detected on ultrasound is associated with impaired cognitive state in the elderly: a population-based study in Wakiso district, Uganda. *J Clin Neurosci.* 2019;68:194–200. 10.1016/j.jocn.2019.06.011 31301929

[14] Wharton SB Simpson JE Ince PG : Insights into the pathological basis of dementia from population-based neuropathology studies. *Neuropathol Appl Neurobiol.* 2023;49(4): e12923. 10.1111/nan.12923 37462105 PMC10946587

[15] Scheltens P De Strooper B Kivipelto M : Alzheimer's disease. *Lancet.* 2021;397(10284):1577–1590. 10.1016/S0140-6736(20)32205-4 33667416 PMC8354300

[16] Dubois B Feldman HH Jacova C : Advancing research diagnostic criteria for Alzheimer's disease: the IWG-2 criteria. *Lancet Neurol.* 2014;13(6):614–29. 10.1016/S1474-4422(14)70090-0 24849862

[17] O'Brien JT Thomas A : Vascular dementia. *Lancet.* 2015;386(10004):1698–1706. 10.1016/S0140-6736(15)00463-8 26595643

[18] Eisenmenger LB Peret A Famakin BM : Vascular contributions to Alzheimer's disease. *Transl Res.* 2023;254:41–53. 10.1016/j.trsl.2022.12.003 36529160 PMC10481451

[19] McArthur JC Hoover DR Bacellar H : Dementia in AIDS patients: incidence and risk factors. multicenter AIDS cohort study. *Neurology.* 1993;43(11):2245–52. 10.1212/wnl.43.11.2245 8232937

[20] Nightingale S Dreyer AJ Saylor D : Moving on from HAND: Why we need new criteria for cognitive impairment in persons living with Human Immunodeficiency Virus and a proposed way forward. *Clin Infect Dis.* 2021;73(6):1113–1118. 10.1093/cid/ciab366 33904889

[21] Montano M Oursler KK Xu K : Biological ageing with HIV infection: evaluating the geroscience hypothesis. *Lancet Healthy Longev.* 2022;3(3):e194–e205. 10.1016/s2666-7568(21)00278-6 36092375 PMC9454292

[22] Sundermann EE Campbell LM Villers O : Alzheimer’s disease pathology in middle aged and older people with HIV: comparisons with Non-HIV controls on a healthy aging and alzheimer’s disease trajectory and relationships with cognitive function. *Viruses.* 2023;15(6):1319. 10.3390/v15061319 37376619 PMC10305373

[23] Barnes RP Lacson JCA Bahrami H : HIV infection and risk of cardiovascular diseases beyond coronary artery disease. *Curr Atheroscler Rep.* 2017;19(5):20. 10.1007/s11883-017-0652-3 28315199 PMC6066370

[24] Guerchet M Mouanga AM M'belesso P : Factors associated with dementia among elderly people living in two cities in Central Africa: the EDAC multicenter study. *J Alzheimers Dis.* 2012;29(1):15–24. 10.3233/JAD-2011-111364 22204904

[25] Yusuf AJ Baiyewu O Sheikh TL : Prevalence of dementia and dementia subtypes among community-dwelling elderly people in northern Nigeria. *Int Psychogeriatr.* 2011;23(3):379–86. 10.1017/S1041610210001158 20716387

[26] Paraïso MN Guerchet M Saizonou J : Prevalence of dementia among elderly people living in Cotonou, an Urban Area of Benin (West Africa). *Neuroepidemiology.* 2011;36(4):245–251. 10.1159/000328255 21677449

[27] Holmes C Cairns N Lantos P : Validity of current clinical criteria for Alzheimer's disease, vascular dementia and dementia with Lewy bodies. *Br J Psychiatry.* 1999;174:45–50. 10.1192/bjp.174.1.45 10211150

[28] Beach TG Monsell SE Phillips LE : Accuracy of the clinical diagnosis of Alzheimer Disease at National Institute on Aging Alzheimer disease Centers, 2005–2010. *J Neuropathol Exp Neurol.* 2012;71(4):266–273. 10.1097/NEN.0b013e31824b211b 22437338 PMC3331862

[29] Akinyemi RO Yaria J Ojagbemi A : Dementia in Africa: current evidence, knowledge gaps, and future directions. *Alzheimers Dement.* 2022;18(4):790–809. 10.1002/alz.12432 34569714 PMC8957626

[30] Paddick SM Longdon A Kisoli A : The prevalence of dementia subtypes in rural Tanzania. *Am J Geriatr Psychiatry.* 2014;22(12):1613–22. 10.1016/j.jagp.2014.02.004 25134968

[31] Feigin VL Abajobir AA Abate KH : Global, regional, and national burden of neurological disorders during 1990-2015: a systematic analysis for the Global Burden of Disease Study 2015. *Lancet Neurol.* 2017;16(11):877–897. 10.1016/S1474-4422(17)30299-5 28931491 PMC5641502

[32] Mills KT Stefanescu A He J : The global epidemiology of hypertension. *Nat Rev Nephrol.* 2020;16(4):223–237. 10.1038/s41581-019-0244-2 32024986 PMC7998524

[33] BeLue R Okoror TA Iwelunmor J : An overview of cardiovascular risk factor burden in sub-Saharan African countries: a socio-cultural perspective. *Global Health.* 2009;5(1): 10. 10.1186/1744-8603-5-10 19772644 PMC2759909

[34] Teunissen CE Verberk IMW Thijssen EH : Blood-based biomarkers for Alzheimer's disease: towards clinical implementation. *Lancet Neurol.* 2022;21(1):66–77. 10.1016/S1474-4422(21)00361-6 34838239

[35] Huber H Blennow K Zetterberg H : Biomarkers of Alzheimer's disease and neurodegeneration in dried blood spots—A new collection method for remote settings. *Alzheimers Dement.* 2024;20(4):2340–2352. 10.1002/alz.13697 38284555 PMC11032540

[36] Dumitrascu OM Qureshi TA : Retinal vascular imaging in Vascular Cognitive Impairment: current and future perspectives. *J Exp Neurosci.* 2018;12: 1179069518801291. 10.1177/1179069518801291 30262988 PMC6149015

[37] Ballerini L Fetit AE Wunderlich S : Retinal biomarkers discovery for cerebral Small Vessel Disease in an older population.Cham: Springer International Publishing,2020. 10.1007/978-3-030-52791-4_31

[38] Cheung CYl Ikram MK Chen C : Imaging retina to study dementia and stroke. *Prog Retin Eye Res.* 2017;57:89–107. 10.1016/j.preteyeres.2017.01.001 28057562

[39] Zhou Y Wagner SK Chia MA : AutoMorph: automated retinal vascular morphology quantification via a deep learning pipeline. *Transl Vis Sci Technol.* 2022;11(7):12. 10.1167/tvst.11.7.12 35833885 PMC9290317

[40] McGrory S Ballerini L Doubal FN : Retinal microvasculature and cerebral small vessel disease in the Lothian Birth Cohort 1936 and Mild Stroke Study. *Sci Rep.* 2019;9(1): 6320. 10.1038/s41598-019-42534-x 31004095 PMC6474900

[41] Sousa RM Ferri CP Acosta D : Contribution of chronic diseases to disability in elderly people in countries with low and middle incomes: a 10/66 Dementia Research Group population-based survey. *Lancet.* 2009;374(9704):1821–30. 10.1016/S0140-6736(09)61829-8 19944863 PMC2854331

[42] Sousa RM Ferri CP Acosta D : The contribution of chronic diseases to the prevalence of dependence among older people in Latin America, China and India: a 10/66 Dementia Research Group population-based survey. *BMC Geriatr.* 2010;10: 53. 10.1186/1471-2318-10-53 20691064 PMC2923155

[43] Alzheimer's Disease International: World Alzheimer Report 2015: the global impact of dementia.2015. Reference Source

[44] Nguyen T Li X : Understanding public-stigma and self-stigma in the context of dementia: a systematic review of the global literature. *Dementia (London).* 2020;19(2):148–181. 10.1177/1471301218800122 31920117

[45] World Health Organisation: Global action plan on the public health response to dementia 2017–2025.2017: Geneva. Reference Source

[46] Robinson L Tang E Taylor JP : Dementia: timely diagnosis and early intervention. *BMJ.* 2015;350: h3029. 10.1136/bmj.h3029 26079686 PMC4468575

[47] Musyimi C Mutunga E Muyela L : The dementia care landscape in Kenya: context, systems, policies and services.2022; 28/11/23. Reference Source

[48] Kamoga R Rukundo GZ Wakida EK : Dementia assessment and diagnostic practices of healthcare workers in rural SouthWestern Uganda: a cross-sectional qualitative study. *BMC Health Serv Res.* 2019;19(1): 1005. 10.1186/s12913-019-4850-2 31881885 PMC6935120

[49] Kakongi N Rukundo GZ Gelaye B : Exploring pathways to Hospital Care for Patients with Alzheimer’s Disease and related dementias in rural South Western Uganda. *BMC Health Serv Res.* 2020;20(1): 498. 10.1186/s12913-020-05365-5 32493309 PMC7268702

[50] Abaasa C Obua C Wakida EK : A qualitative investigation of the psychosocial services utilised by care-givers of patients with Alzheimer's Disease and related dementias in SouthWestern Uganda. *Ageing Soc.* 2023;43(7):1603–1616. 10.1017/s0144686x21001276 37680685 PMC10482049

[51] Asiki G Murphy G Nakiyingi-Miiro J : The general population cohort in rural South-Western Uganda: a platform for communicable and non-communicable disease studies. *Int J Epidemiol.* 2013;42(1):129–141. 10.1093/ije/dys234 23364209 PMC3600628

[52] Mugisha Okello J Nash S Kowal P : Survival of people aged 50 years and older by HIV and HIV treatment status: findings from three waves of the SAGE-Wellbeing of Older People Study (SAGE-WOPS) in Uganda. *AIDS Res Ther.* 2020;17(1): 17. 10.1186/s12981-020-00276-1 32410634 PMC7226937

[53] Hall KS Gao S Emsley CL : Community screening interview for dementia (CSI 'D'); performance in five disparate study sites. *Int J Geriatr Psychiatry.* 2000;15(6):521–31. 10.1002/1099-1166(200006)15:6<521::aid-gps182>3.0.co;2-f 10861918

[54] Nichols E Ng DK Hayat S : Differences in the measurement of cognition for the assessment of dementia across geographic contexts: recommendations for cross-national research. *Alzheimers Dement.* 2023;19(3):1009–1019. 10.1002/alz.12740 35841625 PMC9891734

[55] Ganguli M Chandra V Gilby JE : Cognitive test performance in a community-based nondemented elderly sample in rural India: the Indo-U.S. Cross-National Dementia Epidemiology Study. *Int Psychogeriatr.* 1996;8(4):507–24. 10.1017/s1041610296002852 9147167

[56] Prince M Acosta D Chiu H : Dementia diagnosis in developing countries: a cross-cultural validation study. *Lancet.* 2003;361(9361):909–917. 10.1016/S0140-6736(03)12772-9 12648969

[57] Prina AM Acosta D Acosta I : Cohort profile: the 10/66 study. *Int J Epidemiol.* 2017;46(2):406–406i. 10.1093/ije/dyw056 27154633 PMC5837706

[58] Prince M Ferri CP Acosta D : The protocols for the 10/66 dementia research group population-based research programme. *BMC Public Health.* 2007;7: 165. 10.1186/1471-2458-7-165 17659078 PMC1965476

[59] Dunn G Pickles A Tansella M : Two-phase epidemiological surveys in psychiatric research. *Br J Psychiatry.* 1999;174(2):95–100. 10.1192/bjp.174.2.95 10211161

[60] Üstün, T Kostanjsek N Chatterji S : WHODAS 2.0 - Measuring health and disability.W.H. Organisation, Editor,2010. Reference Source

[61] World Health Organisation: SAGE Well-Being of Older People Study. 2013; [cited 2024 20/2/2024]. Reference Source

[62] Smith SC Lamping DL Banerjee S : Measurement of health-related quality of life for people with dementia: development of a new instrument (DEMQOL) and an evaluation of current methodology. *Health Technol Assess.* 2005;9(10):1–93, iii–iv. 10.3310/hta9100 15774233

[63] Collingwood C Paddick SM Kisoli A : Development and community-based validation of the IDEA study Instrumental Activities of Daily Living (IDEA-IADL) questionnaire. *Global Health Action.* 2014;7(1): 25988. 10.3402/gha.v7.25988 25537940 PMC4275650

[64] Kroenke K Spitzer RL Williams JB : The PHQ-9: validity of a brief depression severity measure. *J Gen Intern Med.* 2001;16(9):606–13. 10.1046/j.1525-1497.2001.016009606.x 11556941 PMC1495268

[65] Spitzer RL Kroenke K Williams JB : A brief measure for assessing generalized anxiety disorder: the GAD-7. *Arch Intern Med.* 2006;166(10):1092–7. 10.1001/archinte.166.10.1092 16717171

[66] World Health Organisation: WHOQOL User Manual.In: *Division of Mental health and Prevention of Substance Abuse. * 2012. Reference Source

[67] Kaufer DI Cummings JL Ketchel P : Validation of the NPI-Q, a Brief Clinical Form of the Neuropsychiatric Inventory. *J Neuropsychiatry Clin Neurosci.* 2000;12(2):233–239. 10.1176/jnp.12.2.233 11001602

[68] Zarit SH Reever KE Bach-Peterson J : Relatives of the Impaired Elderly: Correlates of Feelings of Burden. *Gerontologist.* 1980;20(6):649–655. 10.1093/geront/20.6.649 7203086

[69] de Fátima Ribeiro Silva C Ohara DG Matos AP : Short Physical Performance Battery as a Measure of Physical Performance and Mortality Predictor in Older Adults: A Comprehensive Literature Review. *Int J Environ Res Public Health.* 2021;18(20): 10612. 10.3390/ijerph182010612 34682359 PMC8535355

[70] STRiDE (Strengthening responses to dementia).2022; 27/11/2023. Reference Source 10.1002/dad2.12293 PMC8923343 35317433

[71] Jacobs R Schneider M Farina N : Dementia in South Africa: A Situational Analysis. *Dementia (London).* 2024;23(3):452–475. 10.1177/14713012231183358 37337309 PMC11041067

