# Supplementary material for: Prevalence, aetiology, and service mapping of dementia in rural Uganda. Part of DEPEND Uganda (Dementia Epidemiology, unmet Need and co-Developing Solutions in Uganda)
Source: Wellcome Open Res. 2025 Jan 24;9:544. Originally published 2024 Sep 24. [Version 2] doi: 10.12688/wellcomeopenres.22944.2 (PMC11490832; doi:10.12688/wellcomeopenres.22944.2)
Supplement: Peer review history

# Reviewer report: Reviewer response for version 2

**DOI:** 10.21956/wellcomeopenres.26103.r117741

**Published:** 2025-01-28

**Authors:** Sebastian Walsh

**Affiliations:** 1 University of Cambridge, Cambridge, UK

Thank you to the authors for considering my suggestions and making alterations to the protocol accordingly. Best of luck with this important research.

Is the study design appropriate for the research question?

Yes

Is the rationale for, and objectives of, the study clearly described?

Yes

Are sufficient details of the methods provided to allow replication by others?

Partly

Are the datasets clearly presented in a useable and accessible format?

Not applicable

Reviewer Expertise:

Dementia; public health

I confirm that I have read this submission and believe that I have an appropriate level of expertise to confirm that it is of an acceptable scientific standard.

# Reviewer report: Reviewer response for version 2

**DOI:** 10.21956/wellcomeopenres.26103.r117740

**Published:** 2025-01-28

**Authors:** Ali G Hamedani

**Affiliations:** 1 University of Pennsylvania, Pennsylvania, USA

No further comments.

Is the study design appropriate for the research question?

Yes

Is the rationale for, and objectives of, the study clearly described?

Yes

Are sufficient details of the methods provided to allow replication by others?

Partly

Are the datasets clearly presented in a useable and accessible format?

Not applicable

Reviewer Expertise:

neurology, neuro-ophthalmology, epidemiology

I confirm that I have read this submission and believe that I have an appropriate level of expertise to confirm that it is of an acceptable scientific standard.

# Reviewer report: Reviewer response for version 1

**DOI:** 10.21956/wellcomeopenres.25264.r103383

**Published:** 2024-10-19

**Authors:** Ali G Hamedani

**Affiliations:** 1 University of Pennsylvania, Pennsylvania, USA

This publication contains the details of a protocol aimed at studying the epidemiology of dementia in a rural Ugandan population. The motivations for the study are clear, and the literature review is thorough. The study aims and methods are also clearly presented, with only a few minor clarifications needed:

 1. Regarding the use of cognitive assessments to ascertain dementia, the authors write that this protocol has previously been validated in the 10/66 study but have not been previously used in Uganda. How are they combined to produce a dementia diagnosis according to the 10/66 study protocol, and how will the validity of this be assessed in the current cohort?

 2. The authors describe the stratified sampling strategy for the nested case control study. Aside from the selection probabilities (100% for impaired cognition, 50% for mildly impaired cognition, 15% for unimpaired cognition), how else will the analyses be weighted to be population-representative. For example, if the random 15% of individuals with unimpaired cognition happen to be younger on average than the 85% who were not sampled, how will this be accounted for? Will adjustment for non-response or post-stratification be performed?

 3. For the calculations of overall PAF, how will the analyses account for overlapping risk factors (communality)?

Is the study design appropriate for the research question?

Yes

Is the rationale for, and objectives of, the study clearly described?

Yes

Are sufficient details of the methods provided to allow replication by others?

Partly

Are the datasets clearly presented in a useable and accessible format?

Not applicable

Reviewer Expertise:

neurology, neuro-ophthalmology, epidemiology

I confirm that I have read this submission and believe that I have an appropriate level of expertise to confirm that it is of an acceptable scientific standard.

# Response

**Published:** 2025-01-23

**Authors:** Josephine Prynn

**Affiliations:** University College London, UK

Many thanks for your comments and queries. We have revised the manuscript and present here the responses to your points: 

 1. Regarding the use of cognitive assessments to ascertain dementia, the authors write that this protocol has previously been validated in the 10/66 study but have not been previously used in Uganda. How are they combined to produce a dementia diagnosis according to the 10/66 study protocol, and how will the validity of this be assessed in the current cohort? We have clarified that initially the 10/66 algorithm will be followed, and provided a reference for the publicly available algorithm.

**Response: **We will also be validating the first 25 cases against a clinician diagnosis of dementia. We have re-structured the methods section to make this clearer.

 2. The authors describe the stratified sampling strategy for the nested case control study. Aside from the selection probabilities (100% for impaired cognition, 50% for mildly impaired cognition, 15% for unimpaired cognition), how else will the analyses be weighted to be population-representative. For example, if the random 15% of individuals with unimpaired cognition happen to be younger on average than the 85% who were not sampled, how will this be accounted for? Will adjustment for non-response or post-stratification be performed?

**Response: **If the random sample is not representative of the population, then we can use post-stratification techniques to estimate risk factor prevalence in the underlying population. We have also added a paragraph no handling of missing data and will use sensitivity analysis to explore the potential impact of non-responders.

 3. For the calculations of overall PAF, how will the analyses account for overlapping risk factors (communality)?

**Response:**We will use weighting to account for any communality – the text has been updated to reflect this.

# Reviewer report: Reviewer response for version 1

**DOI:** 10.21956/wellcomeopenres.25264.r103390

**Published:** 2024-10-16

**Authors:** Sebastian Walsh

**Affiliations:** 1 University of Cambridge, Cambridge, UK

Thank you for inviting me to review this exciting and ambitious proposal for essential dementia epidemiological and services assessment in Uganda, as well as exciting, exploratory biomarker work. This proposal is very well written throughout and very clear. I think the methods are sensible and feasible (though ambitious), including care taken over the appropriate use of informant reports to minimise bias. The author group include relevant expertise of Uganda, dementia epidemiology in LMICs, and biomarkers. Below I offer a few more significant thoughts, and then several minor ones.

**MAJOR POINTS:**

1. Aims and objectives: A major challenge from the public health perspective of seeing the new biomarkers (and the associated agenda of pushes for earlier diagnosis) transition from being research-useful to clinically-meaningful is demonstrating that they change overall outcomes for the better. I can see from your planned methods and data sources that it is not feasible to design a cohort study to empirically investigate this. But I wonder if you might consider this question within your planned qualitative work. A patient has a set of symptoms and functional needs which are elicited clinically. How does providing a diagnosis (a ‘precise’ one or otherwise) and how does finding out what their pathology status is affect the treatment and subsequent quality of life of that patient? And do these benefits (assuming there are some) outweigh the opportunity costs of the investment and infrastructure required to deliver the biomarkers routinely? What kind of care pathway is likely to produce the greatest patient/economic benefit at scale? These are questions of relevance globally, but perhaps more so in LMICs where the opportunity costs, even of finger prick or blood-based biomarkers, are likely to be more significant.
2. Missing data: In the analysis plans of parts 1 and 2 I didn’t see any thoughts on how any biases through selection, drop out, incomplete reporting (beyond work with informants) will be mitigated or assessed.
3. Retinal imaging. This is not explained in much detail. I can see that this may be a pragmatic alternative to MRI-based assessment of cerebrovascular burden. But perhaps a little more consideration is needed here as to how valid you think retinal imaging is for assessment of cerebrovascular burden (and its spatial distribution through the brain) and associated dementia risk, and to what extent this is really an exploratory (and pragmatic) approach. And in the analysis plan more detail on how you plan to compare the findings of the retinal imaging with the biomarkers. Is it just amyloid pathology and cerebrovascular disease (as measured by retinal imaging) that you plan to include in the PAF calculation? What about the modifiable risk factors? Age?

**MINOR POINTS:**

Title:

 Suggest that you rephrase this for brevity and impact – ‘Prevalence, aetiology, and service mapping of dementia in rural Uganda. A case-control study nested in DEPEND (Dementia Epidemiology, unmet Need and co-Developing Solutions) Uganda.’

Abstract:

 Suggest paragraph one, “high” is changed to “higher”.

 Suggest paragraph four, abbreviations MRC, URI, LSHTM should be spelled out.

 Suggest final paragraph you add to the sentence “ This paves the way for effective policy strategies for both dementia prevention and support for people with dementia and their families” to include potential for this work to inform research. At the moment you only suggest implications for policy and practice.

Overarching comment:

 You use ‘prevention’ and ‘risk reduction’ somewhat interchangeably through the manuscript and I wonder if you might go through and select terms more deliberately. Access to antiretrovirals for those with HIV might truly prevent dementia. But addressing the Lancet commission risk factors is likely to have a spectrum of effect and perhaps ‘risk reduction’ could be more appropriate in this context.

Introduction:

 In the Dementia Risk Factors section. The sentence: “Together these factors have been estimated to cause around 45% of worldwide dementia cases.” I think the current interpretation of the Lancet commission is that 45% of cases are “attributable” (not ‘caused by’) the combination of these potentially modifiable risk factors.

 In the pathological processes leading to dementia section. The sentence: “AD is considered a progressive neurodegenerative condition which is 60–80% heritable, particularly through the APOE4 genetic pathway ^15^”. You mention some of this later on, but I think you should mention here, that the APOE work (and all genomic dementia work) has been focused primarily on Caucasian/white European populations, and often in clinical datasets which over-select for younger onset (and therefore presumably higher likelihood of genetic association) cases. Some emerging work has suggested the relationships may be different for different ethnic groups.

 This section includes discussion of AD, vascular dementia, and HAND. I think you might add a short additional paragraph after these to mention a) some of the other, rarer causes of dementia (FTD, LBD, reversible forms); and b) that there may be other pathologies which we have not yet fully captured (e.g. recent recognition of the role TDP-43 plays). And other pathological processes, e.g. neuroinflammation, that we haven’t fully understood.

 Somewhere in the introduction it might be good to include a brief description of Uganda as a place. You reference the provenance of several other pieces of research as being from Northern Uganda or Eastern Uganda. But this might not mean so much to the general reader. Perhaps you could include in this paragraph a very brief note on the demographics of the country as a whole – e.g. what is the life expectancy, median age etc. now, what was it 20, 50 years ago? – OurWorldInData.org is a good source for these kind of data; and also some specific information on the region in focus here (e.g. homogeneity of culture, language spoken, typical occupations, HIV prevalence, typical educational background).

Protocol:

 There is mention of stakeholder engagement in part 3 and for dissemination. But I wonder if there is value in initiating this from the outset. Perhaps policymakers and other key stakeholders will have views on what data they need which will add nuance to what data you collect (and how) in parts 1 and 2?

 Part 1. Study setting & population. Forgive my ignorance, but are there any social care facilities or similar entities in this part of Uganda where people would reside if there were perceived challenges to them living well at home? As the authors will know, in HICs a key part of dementia epidemiology is a sampling strategy that includes those not residing in the community, but in health or social care facilities. It may well be that this is irrelevant in this region.

 Part 1. Population. “We aim to recruit all of those with impaired cognition at screening (defined as ≤1.5 SDs below the mean), a random sample of 50% of those with mildly impaired cognition (≤1 to <1.5 SD below the mean), and a random sample of 15% of those with unimpaired cognition (>1 SD below the mean)”. I think all of the greater than/less than signs here are the wrong way round? Please sense check.

 Part 1. Analysis plan. I wonder if you might consider analysing whether the sensitivity and specificity of the screening tools differ by participant characteristic. For example, by stratifying the analysis by sex, by years of education (via a binary cut off), by cognitive score, by age, and/or by language spoken or ethnocultural group (if there is heterogeneity in the region).  

 Part 1. Analysis plan. Please comment on what you plan to do about missing data (e.g. from those not able to complete the 50-90 minute appointment).

 Part 2. Inclusion and exclusion criteria. I think you might present here the expected concordance between the 10/66 algorithm and clinical review, based on existing data from the Sub-Saharan African cohorts for example (e.g. the Nigeria data from the original 10/66 study?). It is currently written as if you are blind to what the concordance is going to be but you are presumably fairly confident that these tools will perform well. There is a big resource implication of validating only for the first 25 cases and validating 200. So you might want to suggest some intermediate steps you could take if the first 25 cases suggest a tweak that needs making to the process for this cohort.

 Part 2. Procedures. Is the Kyamulibwa Research Station easily accessed by the communities you are working with? Is there a risk of bias introduced here by moving from home-based assessments to those where participants must travel?

 Part 2. Procedures. Tobacco smoking is not listed explicitly here, but I assume it is included under ‘vascular risk factors’? Perhaps these could be listed in full for clarity. I also wonder if you might include ‘poverty’ as a potential risk factor to be considered?

 Part 2. Procedures. In part 1 you specific that interviewers will be Luganda-speaking. It would be worth re-stating that here (assuming it is the same) and also that you are sure that all participants living in this area have Luganda as their first language.

 Part 2. Procedures. There is not a timeline provided in this protocol (perhaps a high-level one would be a useful addition?) but one assumes this work will take several years to complete. The blood-based biomarker field for neurodegenerative and related biomarkers is rapidly maturing. The current biomarkers available (AB42/40, P-tau, NFL, GFAP) are quite limited in the breadth of pathology they are designed to estimate, when compared to the neuropathologies indicated by autopsy-based studies (see Nichols Lancet Healthy Longevity 2023 https://doi.org/10.1016/S2666-7568(23)00019-3 for example). But it does seem possible that blood tests, perhaps even via finger prick, might be available for a much broader set of biomarkers might be validated and available soon ( https://www.alzforum.org/news/conference-coverage/nulisa-new-proteomic-method-revamp-biomarker-analysis). I wonder if you might add a line that you will consider broadening your biomarker panel if further biomarkers are validated (and affordable/implementable) in time.

 Part 2. Analysis plan. Again a consideration of the handling of missing data is needed.

 Part 3. Procedures. A little more detail could be given here as to what exactly you are trying to elicit from the interviews (currently quite general topics). The advent of scalable biomarkers, the differing diagnostic criteria proposed by the AA Jack et al. group and the IWG and others, all bring back into sharp focus the idea that we don’t really have a consensus agreement of what Alzheimer’s disease is. And dementia itself means different things in different cultures because what normal cognitive aging is differs across places and time. Cultural beliefs around donation of body tissues and samples can also differ across cultures. It would be good if sufficient space could be given to teasing out some of these foundational questions – ‘what does dementia/Alzheimer’s/mixed disease, growing old with dignity, brain ageing, mean to you? To your community?’. Before the focus on what services are needed which is obviously also important.

Impact of the study and future work

 Tied in with the earlier recommendation to include a paragraph on the study population and the country as a whole. It would be good to include some comment here about how generalisable you think this work is likely to be to the rest of Uganda, and the broader region.

Is the study design appropriate for the research question?

Yes

Is the rationale for, and objectives of, the study clearly described?

Yes

Are sufficient details of the methods provided to allow replication by others?

Partly

Are the datasets clearly presented in a useable and accessible format?

Not applicable

Reviewer Expertise:

Dementia; public health

I confirm that I have read this submission and believe that I have an appropriate level of expertise to confirm that it is of an acceptable scientific standard.

## References

[1] : The prevalence, correlation, and co-occurrence of neuropathology in old age: harmonisation of 12 measures across six community-based autopsy studies of dementia. *Lancet Healthy Longev* .2023;4(3) : 10.1016/S2666-7568(23)00019-3 e115-e125 10.1016/S2666-7568(23)00019-3 36870337 PMC9977689

# Response

**Published:** 2025-01-23

**Authors:** Josephine Prynn

**Affiliations:** University College London, UK

Thank you very much for your considered and thoughtful observations and suggestions. We have revised the submission and hope you will agree that it is stronger for the changes.

**MAJOR POINTS:**

1. Aims and objectives: A major challenge from the public health perspective of seeing the new biomarkers (and the associated agenda of pushes for earlier diagnosis) transition from being research-useful to clinically-meaningful is demonstrating that they change overall outcomes for the better. I can see from your planned methods and data sources that it is not feasible to design a cohort study to empirically investigate this. But I wonder if you might consider this question within your planned qualitative work. A patient has a set of symptoms and functional needs which are elicited clinically. How does providing a diagnosis (a ‘precise’ one or otherwise) and how does finding out what their pathology status is affect the treatment and subsequent quality of life of that patient? And do these benefits (assuming there are some) outweigh the opportunity costs of the investment and infrastructure required to deliver the biomarkers routinely? What kind of care pathway is likely to produce the greatest patient/economic benefit at scale? These are questions of relevance globally, but perhaps more so in LMICs where the opportunity costs, even of finger prick or blood-based biomarkers, are likely to be more significant.
**Response: **Thank you for this observation. We agree that the value in receiving a precise diagnosis is certainly not clear at the moment, although may become so in the future as more effective therapies may become available. The real value of using biomarkers in this study is to gain understanding of the underlying pathological processes leading to dementia, and therefore to understand and promote effective preventative strategies. Furthermore, expanding access to biomarkers aims to help improve equity in diagnostics and drug development so that if or when effective therapy does become available the evidence base comes from diverse populations.
2. Missing data: In the analysis plans of parts 1 and 2 I didn’t see any thoughts on how any biases through selection, drop out, incomplete reporting (beyond work with informants) will be mitigated or assessed.
**Response: ** One of the values of being nested within an established population cohort is that we will have data available describing multiple characteristics of the participants who do not participate, which will help us establish patterns of missingness. Where appropriate, multiple imputation strategies will be used. We have added a statement in the Part 2 analysis plan to describe this.
3. Retinal imaging. This is not explained in much detail. I can see that this may be a pragmatic alternative to MRI-based assessment of cerebrovascular burden. But perhaps a little more consideration is needed here as to how valid you think retinal imaging is for assessment of cerebrovascular burden (and its spatial distribution through the brain) and associated dementia risk, and to what extent this is really an exploratory (and pragmatic) approach. And in the analysis plan more detail on how you plan to compare the findings of the retinal imaging with the biomarkers. Is it just amyloid pathology and cerebrovascular disease (as measured by retinal imaging) that you plan to include in the PAF calculation? What about the modifiable risk factors? Age?
**Response: **The analysis plan has been updated to give more detail about the PAF calculation plans.

**MINOR POINTS:**

Title:

 Suggest that you rephrase this for brevity and impact – ‘Prevalence, aetiology, and service mapping of dementia in rural Uganda. A case-control study nested in DEPEND (Dementia Epidemiology, unmet Need and co-Developing Solutions) Uganda.’

**Response: **Updated

Abstract:

 Suggest paragraph one, “high” is changed to “higher”.

 Suggest paragraph four, abbreviations MRC, URI, LSHTM should be spelled out.

 Suggest final paragraph you add to the sentence “ This paves the way for effective policy strategies for both dementia prevention and support for people with dementia and their families” to include potential for this work to inform research. At the moment you only suggest implications for policy and practice.

**Response: **All done

Overarching comment:

 You use ‘prevention’ and ‘risk reduction’ somewhat interchangeably through the manuscript and I wonder if you might go through and select terms more deliberately. Access to antiretrovirals for those with HIV might truly prevent dementia. But addressing the Lancet commission risk factors is likely to have a spectrum of effect and perhaps ‘risk reduction’ could be more appropriate in this context.

**Response: **Addressed

Introduction:

 In the Dementia Risk Factors section. The sentence: “Together these factors have been estimated to cause around 45% of worldwide dementia cases.” I think the current interpretation of the Lancet commission is that 45% of cases are “attributable” (not ‘caused by’) the combination of these potentially modifiable risk factors.

**Response: **Done

 In the pathological processes leading to dementia section. The sentence: “AD is considered a progressive neurodegenerative condition which is 60–80% heritable, particularly through the APOE4 genetic pathway ^15^ ”. You mention some of this later on, but I think you should mention here, that the APOE work (and all genomic dementia work) has been focused primarily on Caucasian/white European populations, and often in clinical datasets which over-select for younger onset (and therefore presumably higher likelihood of genetic association) cases. Some emerging work has suggested the relationships may be different for different ethnic groups.

**Response: **Done

 This section includes discussion of AD, vascular dementia, and HAND. I think you might add a short additional paragraph after these to mention a) some of the other, rarer causes of dementia (FTD, LBD, reversible forms); and b) that there may be other pathologies which we have not yet fully captured (e.g. recent recognition of the role TDP-43 plays). And other pathological processes, e.g. neuroinflammation, that we haven’t fully understood.

**Response: **Done

 Somewhere in the introduction it might be good to include a brief description of Uganda as a place. You reference the provenance of several other pieces of research as being from Northern Uganda or Eastern Uganda. But this might not mean so much to the general reader. Perhaps you could include in this paragraph a very brief note on the demographics of the country as a whole – e.g. what is the life expectancy, median age etc. now, what was it 20, 50 years ago? – OurWorldInData.org is a good source for these kind of data; and also some specific information on the region in focus here (e.g. homogeneity of culture, language spoken, typical occupations, HIV prevalence, typical educational background).

**Response: **Done

Protocol:

 There is mention of stakeholder engagement in part 3 and for dissemination. But I wonder if there is value in initiating this from the outset. Perhaps policymakers and other key stakeholders will have views on what data they need which will add nuance to what data you collect (and how) in parts 1 and 2?

**Response: **The stakeholder engagement paragraph refers to the whole project, not just Part 3. We have updated the wording to reflect this.

 Part 1. Study setting & population. Forgive my ignorance, but are there any social care facilities or similar entities in this part of Uganda where people would reside if there were perceived challenges to them living well at home? As the authors will know, in HICs a key part of dementia epidemiology is a sampling strategy that includes those not residing in the community, but in health or social care facilities. It may well be that this is irrelevant in this region.

**Response: **We have now this in the first paragraph of Part 1.

 Part 1. Population. “We aim to recruit all of those with impaired cognition at screening (defined as ≤1.5 SDs below the mean), a random sample of 50% of those with mildly impaired cognition (≤1 to <1.5 SD below the mean), and a random sample of 15% of those with unimpaired cognition (>1 SD below the mean)”. I think all of the greater than/less than signs here are the wrong way round? Please sense check.

**Response: **This depends on whether the “less than” signs refer to the score or the standard deviation! Impaired cognition is indicated by a score that is lower than 1.5 SDs below the mean. Therefore a score that is at least 1.5 SDs below the mean (i.e. more than 1.5 SDs).I’ve changed the language to make it less ambiguous.

 Part 1. Analysis plan. I wonder if you might consider analysing whether the sensitivity and specificity of the screening tools differ by participant characteristic. For example, by stratifying the analysis by sex, by years of education (via a binary cut off), by cognitive score, by age, and/or by language spoken or ethnocultural group (if there is heterogeneity in the region). **Response: **Thank you for the suggestion. We are concerned that the stratified analysis would be underpowered, but will assess for bias using a logistic regression modelling approach which we have now outlined in the paper.  

 Part 1. Analysis plan. Please comment on what you plan to do about missing data (e.g. from those not able to complete the 50-90 minute appointment).

**Response: **We have included additional information about the GMS which captures interviewer observation data which helps explain missing data and allows identification of dementia even with an incomplete assessment.

 Part 2. Inclusion and exclusion criteria. I think you might present here the expected concordance between the 10/66 algorithm and clinical review, based on existing data from the Sub-Saharan African cohorts for example (e.g. the Nigeria data from the original 10/66 study?). It is currently written as if you are blind to what the concordance is going to be but you are presumably fairly confident that these tools will perform well. There is a big resource implication of validating only for the first 25 cases and validating 200. So you might want to suggest some intermediate steps you could take if the first 25 cases suggest a tweak that needs making to the process for this cohort.

**Response: **This is a helpful suggestion – we have added a step in which we would attempt to adjust the diagnostic process if concordance is low.   

 Part 2. Procedures. Is the Kyamulibwa Research Station easily accessed by the communities you are working with? Is there a risk of bias introduced here by moving from home-based assessments to those where participants must travel?

**Response: **We have clarified in the text that we will provide transport for those unable to travel independently.

 Part 2. Procedures. Tobacco smoking is not listed explicitly here, but I assume it is included under ‘vascular risk factors’? Perhaps these could be listed in full for clarity. I also wonder if you might include ‘poverty’ as a potential risk factor to be considered?

**Response: **We are assessing for poverty and early life adversity, which I have added to the list, and have clarified which vascular risk factors we are able to assess.

 Part 2. Procedures. In part 1 you specific that interviewers will be Luganda-speaking. It would be worth re-stating that here (assuming it is the same) and also that you are sure that all participants living in this area have Luganda as their first language.

**Response: **Done

 Part 2. Procedures. There is not a timeline provided in this protocol (perhaps a high-level one would be a useful addition?) but one assumes this work will take several years to complete. The blood-based biomarker field for neurodegenerative and related biomarkers is rapidly maturing. The current biomarkers available (AB42/40, P-tau, NFL, GFAP) are quite limited in the breadth of pathology they are designed to estimate, when compared to the neuropathologies indicated by autopsy-based studies (see Nichols Lancet Healthy Longevity 2023 https://doi.org/10.1016/S2666-7568(23)00019-3 for example). But it does seem possible that blood tests, perhaps even via finger prick, might be available for a much broader set of biomarkers might be validated and available soon ( https://www.alzforum.org/news/conference-coverage/nulisa-new-proteomic-method-revamp-biomarker-analysis). I wonder if you might add a line that you will consider broadening your biomarker panel if further biomarkers are validated (and affordable/implementable) in time.

**Response: **Done.

 Part 2. Analysis plan. Again a consideration of the handling of missing data is needed.

**Response: **Done.

 Part 3. Procedures. A little more detail could be given here as to what exactly you are trying to elicit from the interviews (currently quite general topics). The advent of scalable biomarkers, the differing diagnostic criteria proposed by the AA Jack et al. group and the IWG and others, all bring back into sharp focus the idea that we don’t really have a consensus agreement of what Alzheimer’s disease is. And dementia itself means different things in different cultures because what normal cognitive aging is differs across places and time. Cultural beliefs around donation of body tissues and samples can also differ across cultures. It would be good if sufficient space could be given to teasing out some of these foundational questions – ‘what does dementia/Alzheimer’s/mixed disease, growing old with dignity, brain ageing, mean to you? To your community?’. Before the focus on what services are needed which is obviously also important.

**Response: **We agree that exploring the cultural conceptualisation of ageing, brain health and dementia is very important when considering the experiences of people living with dementia and the services available for them, and will be included within the interviews. We have added this more clearly in the text.

Impact of the study and future work

 Tied in with the earlier recommendation to include a paragraph on the study population and the country as a whole. It would be good to include some comment here about how generalisable you think this work is likely to be to the rest of Uganda, and the broader region. **Response: **The text has been updated.
